# Candida Biofilm Eye Infection: Main Aspects and Advance in Novel Agents as Potential Source of Treatment

**DOI:** 10.3390/antibiotics12081277

**Published:** 2023-08-02

**Authors:** Francesco Petrillo, Marica Sinoca, Antonio Maria Fea, Marilena Galdiero, Angela Maione, Emilia Galdiero, Marco Guida, Michele Reibaldi

**Affiliations:** 1 Department of Medical Sciences, Eye Clinic, Turin University, 10126 Turin, Italy; francescopetrillo09@gmail.com (F.P.); antoniomfea@gmail.com (A.M.F.); mreibaldi@libero.it (M.R.); 2 Department of Biology, University of Naples ‘Federico II’, Via Cinthia, 80126 Naples, Italy; m.sinoca@libero.it (M.S.); angela.maione@unina.it (A.M.); marco.guida@unina.it (M.G.); 3 Department of Experimental Medicine, University of Campania “Luigi Vanvitelli”, 81100 Naples, Italy; marilena.galdiero@unicampania.it; 4 NBFC—National Biodiversity Future Center, 90133 Palermo, Italy; 5 Center for Studies on Bioinspired Agro-Environmental Technology (BAT Center), 80055 Portici, Italy

**Keywords:** fungal ocular infection, antimicrobial agents, *Candida albicans*, fungal biofilms, endophtalmitis, keratitis and ocular candidiasis

## Abstract

Fungi represent a very important cause of microbial eye infections, especially in tropical and developing countries, as they could cause sight-threating disease, such as keratitis and ocular candidiasis, resulting in irreversible vision loss. *Candida* species are among the most frequent microorganisms associated with fungal infection. Although *Candida albicans* is still the most frequently detected organism among *Candida* subspecies, an important increase in non-*albicans* species has been reported. Mycotic infections often represent an important diagnostic-clinical problem due to the difficulties in performing the diagnosis and a therapeutic problem due to the limited availability of commercial drugs and the difficult penetration of antifungals into ocular tissues. The ability to form biofilms is another feature that makes *Candida* a dangerous pathogen. In this review, a summary of the state-of-the-art panorama about candida ocular pathology, diagnosis, and treatment has been conducted. Moreover, we also focused on new prospective natural compounds, including nanoparticles, micelles, and nanocarriers, as promising drug delivery systems to better cure ocular fungal and biofilm-related infections. The effect of the drug combination has also been examined from the perspective of increasing efficacy and improving the course of infections caused by *Candida* which are difficult to fight.

## 1. Introduction

Eye infections are one of the most common pathologies in ophthalmology as the eye is excessively exposed to the surrounding environment and comprises intensely vascularized tissues with immunological cellular components that are highly responsive to external stimuli. These infections may affect the external or the internal structures of the eye.

The former is by far the most frequent and results from either the acquisition of a virulent microorganism or the uncontrolled growth of an existing organism due to lowered resistance. The latter is rarer thanks to the defense mechanisms of the eye, which is relatively impermeable to microorganisms; however, they can be caused by trauma, surgery, and blood dissemination [1].

Ocular infections can be classified according to the site of infection and the etiologic agent. Regarding the site of infection, we talk about blepharitis when the eyelids are involved, conjunctivitis when the conjunctiva is involved, keratitis in the case of corneal involvement, uveitis when the uveal tunica is involved, and endophthalmitis when the internal ocular structures are involved [2].

The main etiologic agents are bacteria, viruses, fungi, and parasites. The type of microorganism causing an ocular infection depends on many factors, including geographic location and climate, socio-economic conditions, working conditions, and the presence of additional ocular or systemic diseases [3].

Fungi represent a very important cause of microbial eye infections, and, in some regions, especially in tropical and developing countries, they are among the main causes of blindness, causing sight-threating infections such as keratitis (infection of the cornea that can lead to corneal scarring ad loss of corneal transparency) and endophthalmitis (infection of the internal eye that usually quickly led to irreversible blindness) [4].

*Candida* spp. are the most common fungi isolated in healthy individuals, but a lowering of the eyes’ defense mechanisms could cause severe infections [5]. Infections caused by *Candida* spp. are complicated to treat because of their ability to form a biofilm, a complex of cells embedded in a self-produced extracellular matrix consisting of polysaccharides, proteins or peptides, lipids, and DNA [6].

Most *Candida* species can form biofilms on various types of contact lenses (CL), creating a physical barrier that confers antifungal resistance and CL maintenance solution [7].

This review focuses on fungal eye infections, highlighting the role of *Candida* spp. The main treatments and new strategies to control *Candida* eye infections caused by biofilms are discussed.

## 2. Fungal Ocular Infection

Fungal eye infections are an important cause of blindness worldwide, particularly in developing countries. Different portions of the eye may be affected by mycotic infections, including ocular adnexa, eyelids, lacrimal apparatus, conjunctiva, sclera, cornea, uvea, and internal ocular structures [8]. These infections represent an important diagnostic-clinical problem due to the difficulties in performing the diagnosis and a therapeutic problem due to the limited availability of commercial drugs and the difficult penetration of antifungals into ocular tissues [9].

In this context, the most relevant pathogenic fungi of the eye are *Fusarium*, *Aspergillus* spp., and *Candida* spp., and the most important fungal infections are keratitis and endophthalmitis because of their high risk of blindness [10].

Keratitis is the most common fungal infection of the eye and often results in severe visual impairment. It consists in a purulent ulcerative lesion of the cornea, usually resulting from chronic ocular surface disease, long-term use of topical steroids, prior corneal transplantation, history of corneal trauma with plants or plant material, contact lens wear, systemic immunosuppression, and diabetes [11]. Fungi causing corneal infections can be classified into two main groups: yeasts (e.g., *Candida* spp.), which are more frequent in individuals with chronic ocular surface disease, and filamentous forms (e.g., *Fusarium* spp. and *Aspergillus* spp.), which are more frequent in people wearing contact lens [12].

Endophthalmitis, although rarer, presents devastating effects on the ocular structures, being frequently associated with irreversible reduction in vision. It is defined as the presence and growth of microorganisms within the eye with involvement of the aqueous and vitreous humor and variable inflammatory reaction of the internal ocular structures. It can be classified into endogenous and exogenous forms. The endogenous are complications of fungal infections located in other organs that reach the eye by blood dissemination. Patients most at risk are the immunocompromised, drug addicts, and those on corticosteroids or parenteral or broad-spectrum antibiotic therapy during septicemia [13].

The exogenous, instead, occur as a result of extension of keratomycosis, eye surgery, or penetrating ocular trauma [14]. Interestingly, the eye does not serve as a source of bacteremia and fungemia in the case of infection as the pathogens remain confined to the eye. However, in the case of panophthalmitis, when the walls of the eye are also involved, the infection may spread from the eyeball to the soft tissues adjacent to the orbit [15].

### 2.1. Candida Ocular Infection

Fungi may be part of the normal external ocular flora, and *Candida* spp. is among the most frequent microorganism isolated in healthy individuals, but when ocular defense mechanisms are broken, it could cause sight-threating infections, such as keratitis and endophthalmitis [5]. One feature that makes *Candida* spp. a very dangerous pathogen and difficult to treat is the ability to form biofilm on biotic and abiotic surfaces [16]. Biofilms consist in fungal cells immersed in a polysaccharide extracellular matrix, occasionally represented by the presence of hyphal cells [17].

Regarding keratitis, *Candida* is one of the main etiologic agents. The conditions that predispose to *Candida* infection are the presence of one or more ocular diseases (e.g., insufficient tear secretion, defective eyelid closure), the presence of systemic disease (e.g., diabetes mellitus, immunosuppression), and the presence of pre-existing epithelial defect due to herpes keratitis or contact lenses (CLs) [18]. Moreover, it has been found that *Candida albicans* can adhere to CLs and secrete exopolymers that are almost impenetrable to antibiotics and difficult to eliminate [19].

Symptoms of fungal keratitis (FK) generally occur less acutely than in bacterial keratitis. Patients report foreign body sensation, vision loss, sensitivity to light, and slow onset of increasing pain. The main sign on physical examination usually resembles bacterial keratitis and correspond to purulent discharge, conjunctival hyperemia, corneal epithelial defects, stromal infiltrate, anterior chamber reaction, and hypopyon [20]. In a logistic regression model, serrated margins, raised slough and color other than yellow were found to be independently associated with mycotic keratitis; the probability of fungal infection was 63% if one of these clinical features was present and increased to 83% if all three features occurred [21]. Moreover, the presence of an irregular/feathery border was associated with mycotic keratitis. Although certain clinical signs of infectious keratitis may be associated with a bacterial or fungal etiology, appropriate microbiological tests should be performed at presentation, whenever possible, for a proper diagnosis [22].

With regard to endophthalmitis, the majority are endogenous and occur as a complication of candidemia. Candidemia is a nosocomial infection and an important cause of morbidity and mortality. Every year, Candidemia affects many people in the world and about 50,000 people die [23]. When infection caused by *Candida* occurs, *Candida* spp. can spread to ocular structures: it invades the choroid first, being a very vascularized tissue, and then the other internal eye structures. Therefore, the initial manifestation is usually choroiditis or chorioretinitis, which is characterized by multiple, bilateral, white, well-circumscribed lesions less than 1mm in diameter. These lesions are localized throughout the post equatorial retina and may be associated with vascular sheathing and intraretinal haemorrhages. As the infection worsens, vitritis develops with characteristic exudates with string of pearl appearance and then the inflammation may also involve the anterior segment. Patients may present with blurred or decreased vision if the macular region is involved, with photosensitivity, floaters, and pain arising from anterior uveitis [24].

In the current literature, a distinction is made between *Candida* chorioretinitis and *Candida* endophthalmitis. The latter is appropriately used only in cases of significant vitritis. The term that includes both *Candida* chorioretinitis and endophthalmitis is ocular candidiasis [25]. In studies evaluating the incidence of ocular candidiasis in patients with candidemia, it has been found that it ranges from 2% to 26%, with most cases attributed to chorioretinitis and only 0–6% having endophthalmitis [26,27]. A recent systematic review, based on approximately 7500 examined patients, including more than 1000 identified prospectively, highlighted that endophthalmitis from *Candida* septicemia occurs in less than 1% of the routinely screened [28].

Oude Lashof A.M. et al., in a multicentric study involving 370 patients with candidemia, have found that possible or probable ocular involvement occurred in 16% of subjects, with most cases with chorioretinitis and only 1.6% with endophthalmitis [26].

Son et al. have analyzed all episodes of candidemia in adult patients admitted to Asan Medical Center, South Korea, between January 2014 and May 2017. Patients underwent ophthalmic examinations within 2 weeks after the onset of candidemia. The fundoscopic findings were examined and the ocular infections were classified as endophthalmitis or chorioretinitis and further classified as “proven”, “probable”, or “possible”. During the study period, of the 438 patients with candidemia, only 275 (62.8%) underwent fundoscopic examination within 2 weeks after the onset of candidemia. Of the 275 patients examined, 59 (21.4%) had ocular involvement, of which 8 (2.9%) had endophthalmitis and 51 (18.5%) had chorioretinitis [29].

The main risk factors for ocular candidiasis are hospitalization with a history of recent major gastrointestinal surgery, bacterial sepsis, systemic antibiotic use, immunomodulatory therapy, intravenous drug abuse, indwelling catheter, general debilitation, organ transplantation, and prolonged neutropenia [30].

Following a comparison between people with and without ocular engagement, Son et al. reported that the use of corticosteroids during the previous 6 weeks, chemotherapy during the previous 6 weeks, neutropenia during the previous 2 weeks, *C. albicans,* and persistent candidemia were, respectively, (45.8% vs. 30.1%), (39.0% vs. 24.1%), (16.9% vs. 6.5%), (61.0% vs. 37.0%), and (33.9% vs. 16.7%). In contrast, *C. glabrata* (8.5% vs. 31.9%) was significantly less frequent among patients with ocular involvement. Fungemia caused by *C. albicans*, persistent candidemia, and neutropenia during the previous 2 weeks were factors independently associated with ocular involvement, whereas *C. glabrata* was inversely associated with ocular manifestations [29].

Cases of exogenous *Candida* endophthalmitis are rare and follow surgery or trauma. Usually, the inflammation first affects the anterior chamber and then the vitreous humor. *Candida parapsilosis* seems to be the most common species in post-surgery infections, as it easily survives in fluids used during surgeries and prosthetic material [25].

In 1983, an outbreak in the USA affected 13 patients in different states, following cataract extraction or intraocular lens implantation. This outbreak followed the introduction of a new brand of balanced salt solution (BSS) used as an intraoperative ophthalmic irrigation solution. This product was subsequently recalled because of intrinsic fungal contamination [31].

The Table 1 summarizes the characteristics of the main types of fungal eye infections.

### 2.2. Epidemiology of Candida spp.

*C. albicans* is the most frequently detected organism in *Candida* infections, although some studies report an important increase in non-*albicans* *Candida* [32].

Ranjith et al. have analyzed about 50 fungal isolates from patients with keratitis, endophthalmitis, and orbital cellulitis. They used the Vitek-2 system and sequencing of the ITS1-5.8S-ITS2 regions of the rRNA gene, followed by phylogenetic analysis for phenotypic and genotypic identification [33]. The commonly isolated species was *C. parapsilosis* (in 21 samples), followed by *C. viswanathii* (in 9 samples). This study reverses a trend confirmed in previous studies, in which it was observed that the predominant species in ocular candidiasis is *C. albicans*. This is probably because in those studies the analysis relied on phenotypic methods such as hyphal formation and thus non-*albicans* species were not adequately identified [33].

Belanger N.L. et al. characterized 38 yeast isolates collected from patients with endophthalmitis or keratitis at the Massachusetts Eye and Ear from 2014 to 2021. They observed that for both endophthalmitis and keratitis, more than 50% of isolates were *C. albicans*. Subjects with endophthalmitis had *C. dubliniensis* as the second most common yeast. In subjects with keratitis, the second most common species was *C. parapsilosis*. They also noted that non-*albicans* species have less predictability in their susceptibility to antifungal agents, which means that these infections may be more difficult to treat [34].

Motukupally et al. defined the microbiological profile of patients with ocular candida infection. Out of a total of 42 patients, 29 were affected by keratitis, 12 by endophthalmitis and 1 by orbital cellulitis. Forty-two *Candida* isolates were analyzed using the compact Vitek 2 system, and it was found that the predominant species was *C. albicans* (in 12 cases of keratitis, 4 of endophthalmitis, and 1 of orbital cellulite), followed by *C. parapsilosis*, *C. guilliermondii*, *C. ciferrii*, *C. glabrata*, and one block of *C. tropicalis* [35].

Ueda et al. have included in their study, between 2010 and 2016, non-neutropenic patients with candidemia, aged over 17 and hospitalized in 1 of 15 medical centers in Japan. Patients suffering from candidemia were subjected to ophthalmological examination, and it was found that the prevalence of ocular candidiasis was 19.5%. The most commonly isolated *Candida* species was *C. albicans* (77.9%), followed by *C. glabrata* and *C. parapsilosis* (8.4% each), *C. tropicalis* (3.9%), and *C. krusei* (0.6%) [36].

Abe et al. reviewed the medical records of patients with candidemia from January 2012, for about 5 years, at the Toranomon Hospital (Tokyo). Then, they assessed ocular fungal load, levels of inflammatory cytokines and chemokines, and levels of inflammatory cells in mice infected with *Candida albicans* and non-*albicans* species [37]. While under study, 99 patients with candidemia were examined by ophthalmologists, and 20 were found to have ocular candidiasis. Cases with candidemia were excluded if patients died before the examination of blood cultures and if, due to the serious condition of the patient, it was not possible to perform the analysis of the ocular fundus. Blood culture specimens were analyzed using Bactec 9240 and Bacterc FX systems (Becton, Dickinson and Company, Sparks, MD, USA), and all *Candida* species were isolated on Sabouraud dextrose agar at 35 °C. Species were identified by a Vitek or Vitek 2 system (bioMérieux, Marcy l’Etoile, France) for all *Candida* negative for the germ-tube test. As an additional support for species identification, some regions of the rRNA gene were sequenced [37]. Female mice were infected with *Candida albicans*, *Candida parapsilosis*, and *Candida glabrata* (both reference strains and clinically isolated strains) and were suppressed three days after infection, for eyeball collection. It was observed that although *Candida parapsilosis* was the most common pathogenic microorganism detected, in the case of candidemia, *Candida albicans* was considerably related to ocular candidiasis. In vivo, tests showed that in mice there was an increased level of both fungal load and mediators of inflammation during *C. albicans* infection. In addition, following *C. albicans* infection, the levels of neutrophils and ocular monocytes were also much higher compared with non-*albicans* infections. Histopathological analysis revealed increased invasion of ocular tissues by *C. albicans*, which invades the retina through hyphae and in one specimen also invaded the vitreous humor. These results suggest that *C. albicans* compared to *Candida* non-*albicans* is the species most associated with ocular candidiasis due to its increased ability to invade, induce inflammatory mediators, and recall monocytes and neutrophils [37].

### 2.3. Diagnosis

Often, in the case of fungal eye infections, it is difficult to make a clinical diagnosis, isolate the pathogen, and identify effective treatment. The most difficult part of the diagnosis is the microbiological detection of the etiological agent in clinical samples. To prevent serious complications, it is important that diagnosis and treatment are performed immediately [8].

With regard to mycotic keratitis, the diagnosis is based on clinical suspicion, which is determined by the presence of signs and symptoms of keratitis and the typical risk factors and then confirmed by microbiological analysis. The gold standard method for etiological diagnosis consists of corneal scraping, using a kimura spatula or blade, followed by cultures by direct seeding in solid and liquid media [38]. Fungi associated with eye infections are usually fast growing saprophytic fungi that can grow on media, such as blood and chocolate agar, traditionally intended for bacteria. However, in the case of strong suspicion, specific media can be used, such as Sabouraud agar, which allows a presumptive and early identification [3]. Growth in culture is considered significant if the same growth is obtained (i) on more than one occasion, (ii) on the ‘C’ streaks on more than one culture medium, or (iii) on one solid or in one liquid medium with direct microscopy of corneal material revealing the presence of yeast cells [16]. The material collected by corneal scraping can also be used for direct microscopic examination. This method is very important as it allows the quick identification of the presence of fungi and guides the empirical therapy before the precise identification of the pathogen through the culture techniques [39]. The main differential stains used for direct microscopic examination are Gram stain (to evaluate the presence of bacteria, fungi and parasites, and to distinguish Gram-positive from Gram-negative bacteria), Lactophenol Blue stain (to detect early the presence of fungi), Calcofluor white + potassium hydroxide stain (to detect early the presence of fungi and *Acanthamoeba*, the latter if a fluorescence microscope is available) [3]. Moreover, molecular methods, in particular PCR, could also be used for etiological diagnosis. This test has the great advantages that it requires only a small quantity of sample, is very rapid, and permits the molecular identification of isolated fungi. However, the most important disadvantages are the easy contamination, the possible amplification of the genome of dead microorganisms, the possible cross-reactivity with different microorganisms, and the cost [38]. When corneal scrapings yield negative results, it is possible to perform a corneal biopsy [19]. In vivo confocal microscopy is a new non-invasive diagnostic tool that could be helpful in the case of fungal keratitis. It allows in vivo examination of the cornea with a magnifications of up to ×200 to ×500 and an increased image contrast, which can enable a presuntive identification of the microbial agent and infection monitoring [40]. Vaddavalli et al. have evaluated the role of confocal microscopy in patients with proven fungal keratitis. Out of 93 microbiologically diagnosed cases of fungal keratitis, 83 were also identified correctly on confocal microscopy with a sensitivity of 89.2% (95% CI, 83–95.5) and a specificity of 92.7% (95% CI, 85.9–99.6). The criterion used for the identification of fungal filaments on confocal microscopy was the presence of highly reflective, septate, double-walled filaments varying in size between 3 and 8 microns [41]. The main limitation to its current use are the cost of the instrument and the lack of experience.

With regard to ocular candidiasis, the diagnosis is usually clinical, based on the presence of typical lesions of chorioretinitis or endopthalmitis in the appropriate clinical context, such as patients with disseminated *Candida* infection or significant risk factors, and then it may be confirmed by microbiological analysis [42]. The gold standard method for etiological diagnosis consists of culture exams. The biological sample should be collected by aqueous sampling or vitreous sampling. However, the collection of vitreous humor is considered the most reliable and most sensitive method for fungal endophtalmitis diagnosis [43]. Vitreous humor samples can be obtained either via posterior chamber needle aspiration or via therapeutic vitrectomy, the latter method being the most reliable for fungi identification [44]. Anyway, the yield of positive cultures from vitreous samples is usually lower in cases with fungal endophthalmitis than in cases with bacterial endophthalmitis [14]. Direct microscopic examination, with the use of differential stains, such as Gram, Lactophenol Blue, Calcofluor white + potassium hydroxide, and Giemsa, may lead to a rapid presumptive diagnosis and guides the empirical therapy. Intraocular fluids require (before direct examination) cytocentrifugation [3]. In the case of endogenous endopthalmitis it is recommended to perform blood cultures. However, positive *Candida* blood cultures occur in only 50–75% of patients with Candida endogenous endophthalmitis, presumably because some patients only have transient or intermittent fungemia [45]. Lastly, detection of *Candida* DNA in intraocular fluid can be carried out using PCR assays. This method has been shown to be sensitive and rapid and overcomes the limitations of vitreous fluid culture [46]. Moreover, it seems that the efficacy of PCR performed from aqueous humor is similar to PCR performed from vitreous humor, with the first being very much easier to collect. However, the number of pathogens that can be simultaneously searched for is limited, and that constitutes an important problem, above all for ocular specimens, which are in most cases irreplaceable [47].

The Table 2 ssummarizes some diagnosis strategies for the main eye diseases.

## 3. Role of Biofilms in Ocular Infection

A strategy commonly used by microorganisms to become resistant to antimicrobials is the ability to form biofilms [48].

Biofilm protects microbes from hostile environmental conditions and at the same time microbes show metabolic cooperation, gain AMR phenotypes, and show altered expression of virulence genes and virulence factors. Microbial biofilms are involved in many diseases, as they represent a favorable environment for the growth of microorganisms and the development of virulence. Biofilms are clusters of microorganisms embedded in a self-produced matrix of extracellular polymeric substances (EPS) [49]. Fungal biofilms are a group of cells immersed in an extracellular matrix (ECM) with the ability to adhere to each other and on different surfaces [50,51,52,53]. Biofilm formation is a feature of *Candida* spp. and, depending on the species considered, that involves different phases: an initial attachment to a surface, filamentation, cell to cell interactions, biofilm maturation, and dispersal towards new sites helping them to escape from compounds or drugs used for biofilm disruption [54,55]. In the case of eye infections biofilms are usually formed on ocular abiotic surfaces like contact lenses, scleral buckles, intraocular lenses, and sutures. Current studies show that biofilm formation may take place immediately on the biotic surfaces of the eyes, such as mucosal surfaces (e.g., cornea). The first step is very important, and microorganisms must be sufficiently close to the surface to which they must adhere. Both attraction and repulsion forces intervene and depend on the size of the microorganism, the distance from the surface, and the hydrophobicity of the surface itself. For surfaces wetted by fluids, such as conjunctiva or contact lenses, these forces also depend on the presence of solutes within them [56]. Consequently, clinically, biofilm diseases can be extremely difficult to treat because of their resistance to treatments, host immune defenses, and also persistence on mucous surfaces. Biofilm is also observed on medical devices such as catheters, implants, heart valves, intraocular lenses, orthopedic devices and contact lenses [57].

Biofilms can be monomicrobial and polymicrobial. Polymicrobial biofilms are formed by different bacteria, different fungi or by bacteria and fungi together and they are more difficult to treat than monomicrobial biofilms and related planktonic cells [58,59]. The use of contact lenses is an important risk factor for microbial keratitis. Water content, hydrophobicity, and roughness are aspects that influence how different microorganisms interact with different materials. The main factor of virulence is the ability of microorganisms to form biofilms and produce adhesion factors that facilitate their permanence on lenses and containers [60]. Although FK accounts for only 1.5% of all cases of keratitis in contact lens users, the presence of fungi and the subsequent formation of biofilm in contact lenses poses an increasing threat to public health, especially for the ability of fungi to form polymicrobial biofilms, difficult to eradicate [7]. Most of *Candida* spp. are biofilm producers; this is an important factor associated with virulence and resistance to antifungals. Inappropriate manipulation of contact lenses facilitates the entry of infectious agents into the lenses.

According to the literature, the risk of complications resulting from the use of soft contact lens subtypes is higher than that resulting from the use of rigid contact lenses [61]. It has been discovered that several tear proteins, such as albumin, lysozyme, and fibronectin, increase the adhesion of *Candida* to contact lenses [62,63]. Some studies have observed the existence of a positive correlation between the adhesion to *Candida* and the level of water in contact lenses. Both *Fusarium* and *Candida* are able of forming biofilms on different types of lenses. The presence of biofilm has decreased the effectiveness of contact lens solutions [64].

Mukherjee et al. have conducted an in vitro study on contact lenses inoculated with keratitis-isolated *Fusaria* and observed that biofilm capacity is a critical factor for pathogenesis. Furthermore, the biofilm formed by *Fusarium* isolates showed higher drug resistance in comparison to planktonic cells [65].

Fritsch et al., 2020 [16] studied the formation of biofilms on different types of contact lenses materials by *C. albicans* and *C. krusei* and the relative development of preventive or reductive treatment to avoid eye infections among contact lenses users. They found that both *C. albicans* and *C. krusei* strains were able to form biofilms on contact lenses, confirming the fact that they are a suitable surface for *Candida* spp. adhesion and growth and that the formation of biofilms with greater metabolic activity and greater biomass was noted for soft contact lenses by both the species, considering their surface hydrophobicity.

However, current research has shifted the interest to more in vivo and ex vivo examples of fungal biofilm association because there are some limitations associated with the use of in vitro systems for the study of biofilm infections. For example, the microenvironment of the biofilm influences the structure and morphology of the biofilm [66]. Ex vivo studies make use of whole corneas. Due to the limited availability of human corneas, animal corneas are often used, and therefore interspecies variation is a major problem with ex vivo studies [67].

Ranjith et al., in their study, reported that *S. aureus* and *S. epidermidis* isolated from patients with endophthalmitis and *C. albicans* isolated from a patient with keratitis, form polymicrobial biofilms both in vitro using tissues culture plates and ex vivo using human cadaveric cornea as the substratum for biofilm formation. Polymicrobial biofilms showed an increase of several fold resistance to antimicrobial agents than monomicrobial biofilms and planktonic cells.

In conclusion, biofilm formation at the level of intraocular structures or on devices such as contact lenses is the main therapeutic obstacle. Biofilms, especially polymicrobial, being formed by different microorganisms, make the use of conventional antibiotics and antifungals ineffective. Added to this is the fact that when microorganisms acquire, at both genotypic and phenotypic levels, biofilm formation, they also usually acquire new characteristics in terms of resistance to antimicrobial substances.

## 4. Current Treatment Options

Fungal ulcers often have worse outcomes than bacterial ulcers due to the reduced ocular penetration and efficacy of antifungal drugs and the difficult diagnosis of this condition [20]. The management consist of medical therapy alone or in combination with surgical treatment [68].

The antifungal drugs are the backbone of medical therapy and the most widely used ones are classified in four groups: polyenes, azoles, pyrimidines, echinocandins.

Among the polyenes, the most commonly used compounds are amphotericin B and natamycin. Among the azoles, the most frequently used molecules are voriconazole, fluconazole, econazole, itraconazole, miconazole, and ketoconazole. Among the echinocandins, caspofungin and micafungin have been successfully used in the treatment of mycotic keratitis. Finally, among the pyrimidines, the most frequently used is flucytosine [22]. The main routes of administration are topical, oral, and intravenous. The subconjunctival route is not frequently used due to the toxicity and pain induced, while the intrastromal route is reserved to severe keratitis and recalcitrant to topical treatment [20]. Topical drugs are the mainstay of treatment because they can reach high concentrations with few systemic side effects. The main formulations are natamycin 5%, amphotericin B 0.15–0.3%, voriconazole 1–2%, econazole 1%, ketoconazole 1–2%, fluconazole 1%, itraconazole 1%, and flucytosine 1% [22]. Topical natamycin 5% is the only that is FDA-approved and commercially available in the United States, while the others need galenical preparation. The majority of topical compounds are fungistatic rather than fungicidal, while amphotericin B has shown fungicidal activity, particularly against *Candida albicans*, but the fungicidal activity is less predictable against molds [19]. The treatment is started once the specimen has been taken and is chosen empirically considering the clinical presentation and the direct microbiological examination. Topical natamycin 5% is usually chosen as initial therapy. In the case of severe infection additional antifungal compounds can be added (e.g., amphotericin B or voriconazole) and in the case of *Candida* isolation topical amphotericin B or topical fortified voriconazole 1% are preferred [22]. In Mycotic Ulcer Treatment Trial (MUTT) was investigated the efficacy of topical natamycin versus topical voriconazole 1% in treatment of fungal corneal ulcers. While visual improvement in ulcers secondary to *Fusarium* was significantly increased in patients who received topical natamycin, among non-*Fusarium* ulcers, no difference in visual acuity was found [69]. With regard to systemic medication, azole antifungals class represent the mainstay. Although the Mycotic Ulcer Treatment Trial 2 (MUTT 2) concluded that oral voriconazole made no difference in the treatment of severe filamentous keratitis and in the incidence of corneal perforation, it is usually prescribed in the case of scleral or internal ocular structure involvement [69]. Medical therapy also includes cycloplegics for the management of pain and inflammation caused by anterior uveitis and antibiotics to prevent secondary bacterial infection. On the contrary topical corticosteroids are contraindicated. Finally therapeutic keratoplasty is usually indicated in the case of impending perforation, perforation, or unfavorable evolution to medical therapy [18].

The management of intraocular candidiasis depends on clinical context. Patients who only have chorioretinitis, without involvement of the vitreous, often respond well to systemic antifungals alone [70]. In the case of endophthalmitis (with vitreous involvement) it is usually necessary to add intravitreal therapy or surgical therapy. If patients have concurrent candidemia, it is important to treat both the systemic and the ocular condition which is essential due to the high rate of morbidity and mortality associated with candidemia [71]. Local endophthalmitis (exogenous), without concomitant candidemia, has been treated with vitrectomy and intravitreal injections alone [70]. The main drugs used for systemic antifungal therapy are fluconazole, voriconazole, liposomal amphotericin B and flucytosine [14]. The mainstay of intravitreal therapy is amphotericin 5–10 µg/0.1 mL or voriconazole 100 µg/0.1 mL, and the injection can be can be repeated in recfractory cases after 72 h [14]. With regard to surgical therapy, *Pars plana* vitrectomy may be useful both as a diagnostic tool, allowing the identification of the microbes, and as a therapeutic tool, by debulking the pathogen load and treating surgical complications that result from the infection [72].

Recently, the Royal College of Ophthalmologists’ Professional Standards Committee and the Intensive Care Society and the Faculty of Intensive Care have approved guidelines for ocular candidiasis treatment, based on clinical manifestation [73]. In the case of choroiditis, as the lesions are localized outside the blood–retinal barrier (BRB), systemic amphotericin B (which is effective against *Candida* but poorly crosses the BRB) is indicated. In the case of chorioretinitis, as the lesion is localized inside the BRB, the amphotericin B (even in lipophilic form) will not achieve a local minimum inhibitory concentration, so fluconazole (oral or intravenous), which is demonstrated to cross well the BRB, is needed; then, it is necessary to watch daily the patient to ensure that infection does not progress. Finally, in the case of endophthalmitis (when the lesion involves the vitreous), intravitreal amphotericin plus oral fluconazole are indicated [73].

The Table 3 summarizes the therapy used to treat fungal eye infections.

## 5. Antifungal Resistance in Ocular Infections

Antimicrobial resistance has become one of the major health threats of the 21st century. The emergence of MDR organisms poses a threat to patient care because the only agents to which many microorganisms are now sensitive are toxic, expensive, and not widely available as topical drugs [74].

Fungi infect billions of people and kill about 1.5 million people a year, comparable to the mortality rates of widely recognized diseases, such as tuberculosis or malaria [75]. The prevalence of serious diseases caused by fungi has increased in recent decades due to the increase in the number of immunocompromised individuals, including cancer patients, organ transplants, individuals infected with HIV, and the increase in the elderly population [76]. Despite the harmful effect of fungi on human health, only a few classes of antifungals are currently available to treat these life-threatening infections. Largely, the development of new antifungals has been slow due to the eukaryotic nature of fungal cells, problems of permeability of compounds through the cell wall and fungal membrane and limited interest of the industry in the development of new antifungals [77].

Over the years, *Candida* spp. have developed resistance to azoles, polyenes, and other common antifungals, contributing to the emergence of strains resistant to antifungal agents. This resistance is acquired mostly due to the overuse of drugs and the mechanisms of virulence developed by microorganisms [76]. There are numerous mechanisms of resistance to antifungal drugs, including alteration or overexpression of the drug’s target, over-regulation of multidrug transporters, and activation of responses to cellular stress [76].

An important factor of virulence, but not the only one, that contributes to resistance is the formation of biofilms. It is likely that the greater resistance associated with biofilms is multifactorial and due to the exopolysaccharide matrix that limits the penetration of antimicrobials in biofilm, eDNA and polysaccharides (present in biofilm) that can trap molecules of opposite charge, the presence of persistent cells in biofilms (a population of cells whose growth rate is zero or extremely slow), and efflux pumps that allow bacterial cells to pump out toxins and drugs [78].

## 6. Novel Strategies to Control Biofilm-Associated Ocular Infections

The administration of ocular drugs remains a great challenge for researchers and ophthalmologists due to the limits of conventional delivery systems, such as eye drops or oral/venous administration, that reduce the efficacy of medical therapy [79]. In this context the major problems are presence of physiological barriers, biofilm formation, enzymatic degradation, poor targeting efficiency, poor penetration, low retention time, low bioavailability, and antifungal drug resistance. Emerging strategies in ocular delivery systems try to overcome the limits of the extraocular and systemic routes.

The use of polymer biomaterials can prolong treatment release and reduce the frequency of administration. The versatility of biopolymers and synthetic polymers opens the door to many types and forms of biomaterials used as drug delivery vehicles to treat ocular diseases. Within the field of micro and nanotechnology, there are several mechanisms of drug delivery, such as microparticles, nanoparticles (NPs), micelles, and liposomes. These new transport mechanisms have the advantage of being less invasive, as well as the ease of injection through small-gauge needles. These have also been explored for incorporation into drug-eluting contact lenses to facilitate topical delivery [80]. Nanoparticles allow us to obtain better eye retention, penetration, and bioavailability of drugs; instead, to avoid the loss of the drug through drainage and prolong the time of contact of the drug with the affected area, drug-releasing contact lenses are a viable alternative [81]. Different nanosystems for drug delivery to the eye can be found in the literature as liposomes, polymeric NPs, lipid NPs, niosomes, micelles, dendrimers, and nanofibers [82,83].

In nanoparticle (NP)-mediated drug delivery, liposomes are the most used drug transporter, and the only NP mechanism already agreed by the FDA for clinical use, owing to their beneficial physicochemical features and exceptional biocompatibility. Liposomes are spherical vesicles with a phospholipidic double layer, thanks to which the drug is encapsulated inside them, and its release is controlled. Liposomes are capable of carrying hydrophilic and hydrophobic substances. They have the advantage of being biodegradable, biocompatible and non-toxic nanosystems [84]. Since amphotericin B has the disadvantage of poor penetration in cells and is also toxic to the epithelium, new strategies have been developed to improve its effectiveness. In fact, liposomes can improve the affinity between antifungal molecules and sterols in the fungal cell [85].

The most recent research regarding polymeric nanoparticulate materials for ocular drug delivery, including micelles, dendrimers, cyclodextrins, and polymeric vesicles, will be explored, with all of them administered via ophthalmic drops [86].

To reduce fluconazole (FLZ) adverse effects, increase drug activity, and maintain drug delivery, many techniques have been used. M. Almehmady et al. developed an optimized FLZ-polymeric nanoparticle formulation and charged it into two different ophthalmic formulas. The compound showed higher antifungal activity against *Candida albicans* when compared to the pure drug, confirming a promising drug delivery system in the treatment of deep ocular fungal infections [87].

Khan et al. [88] developed multifunctional gallic acid (GA), phytomolecule-covered zinc oxide nanoparticles (ZN), and phytomolecule-covered zinc oxide nanoparticles + gallic acid + tobramycin (ZGT)-coated contact lenses. Coated contact lenses had antibacterial, antifungal, and antibiofilm activity against several multi-resistant species that cause bacterial keratitis (e.g., *Staphylococcus aureus*, *Pseudomonas aeruginosa*) and against FK (e.g., *Candida albicans*, *Aspergillus fumigatus*). Coated lenses showed good antioxidant, biocompatibility, and wettability characteristics.

Alakkad et al. [89] have speculated that MET (molecular envelope technology (N-palmitoyl-N-monomethyl-N,N-dimethyl-N,N,N-trimethyl-6-O-glycolchitosan) nanoparticles enter inside *C. albicans* biofilms in vitro and that MET-AmB eye drop formulations may provide a superior therapeutic outcome in biofilm-associated ocular fungal infections. In addition, they supported that a MET-AmB formulation was a more active antifungal formulation when compared to the drug alone.

Posaconazole (PSC) is the second-generation thiazole agent with the widest spectrum among anti-fungal agents and is considered an important anti-fungal option in the treatment of both anterior and posterior ocular fungal infections with its broad spectrum and low minimum inhibitory concentration (MIC). PSC is also a highly lipophilic molecule, insoluble in water and a relatively high-molecular-weight compound, which is likely to limit its ocular bioavailability.

Durgun et al. [90] developed a novel delivery system of PSC-micelles with safe anti-fungal activity which provides delivery via the ocular route.

Gallic acid is a phenolic acid with antioxidant and antimicrobial properties [91]. Metal-based anti-infective agents have attracted the attention of researchers due to their antimicrobial properties. Although many metals have antifungal activity, they should be used with caution because of their toxicity.

He et al. [76] observed that certain metals and enzymes could degrade exopolysaccharides and act on planktonic cells and mature biofilms of *Candida*, without causing adverse effects. In particular, their study has focused on lyticase and gallium ions involved in nanosystems (MLPGa). The potential antifungal mechanism of MLPGa depends on the polysaccharide-specific degradation, intrinsic ROS production, and metabolic interference, including up-regulating of antioxidant-related genes, exopolysaccharide-related genes, and down-regulating of iron ion-utilization-related genes, fungal/biofilm development-related genes, and virulence genes. The use of MLPGa gave good therapeutic results, in vivo, in the infected mouse eyes of the *C. albicans*-infected FK model [92].

In another study, Ahmed et al. (2022) [93] developed ocular oleylamine-covered FTN-loaded olaminosomes using an ethanol injection system to treat candidiasis with in vivo corneal test. Fenticonazole nitrate (FTN) is in the family of imidazoles firstly formulated, including terpesomes and cerosomes. They observed that the FTN-loaded optimum formula considerably supported the antifungal activity both in vitro and in vivo of FTN on the ocular surface for longer period in comparison to the FTN suspension, confirming the hypothesis that olaminosomes could be considered as promising transporters to improve the ocular transport of the drug fenticonazole nitrate.

**Figure 1:**
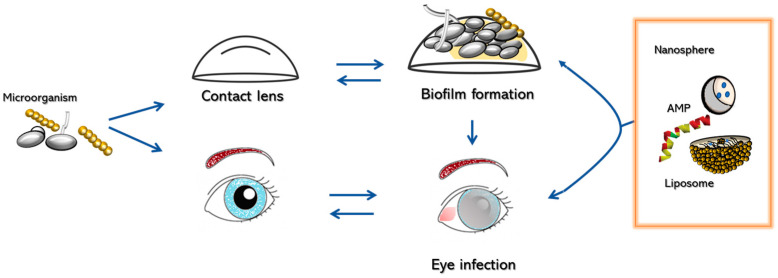
Nanosystems and alternative compounds for the treatment of fungal infections.

In addition to the use of nanosystems in the treatment of keratitis, one of the prospects is represented by using alternative compounds as drugs, such as natural compounds or antimicrobial peptides (Figure 1).

Research and development of new drugs is aimed at the study of phytoextracts, which contain a pool of bioactive compounds with different biological activities, including antimicrobial ones. It has been shown that phenolic compounds, isolated from natural sources, have shown anticandidal activity, even if knowledge on the biological properties of various medicinal plants remains limited.

*Orobanche crenata* (OCLE), a parasitic plant particularly widespread in the Mediterranean area, has shown the ability to inhibit the growth of numerous bacteria and *Candida* spp. [94]. D’Angeli et al. have shown that OCLE can inhibit the growth of *C. albicans* and *C. glabrata* but does not cause death (it has a fungistatic effect). Since the strain of *C. glabrata* used by them under experimental conditions did not form biofilms, they evaluated the effect of OCLE on the formation of biofilms by *C. albicans*. Biofilm formation is stimulated at low concentrations and inhibited at high concentrations. This could be the consequence of an ormetic mechanism [95].

Interest in the pharmacological applications of cannabinoids is largely increasing in a wide range of scientific areas. Research on their potential role in eye diseases has now intensified, many of which are chronic and/or disabling and require new alternative treatments. However, due to the unfavorable physico-chemical properties of cannabinoids and adverse systemic effects, along with ocular biological barriers to local drug administration, drug delivery systems are needed. As one of the symptoms of keratitis is pain, modulation of the endocannabinoid system has become the main approach to relieve suffering and inflammation. Despite the interesting pharmacological properties, cannabinoids show numerous side effects especially when administered systemically.

An interesting approach is the use of nanocarriers, including polymer NP and carbon nanotubes. Nanostructures composed of hydrophobic materials seem to be the best option to carry cannabinoids due to their lipophilic character [96].

Di Onofrio et al. evaluated the ability of two natural mixtures, the fermented extract of *Allium sativum* (BGE) and the oil extract of cannabinol (CBD), to avoid biofilm formation and eradicate mature biofilms of *P. aeruginosa* (clinical strains) on soft lenses, compared to a multipurpose solution on the Italian market. The study showed that BGE and CBD have a good effect on inhibiting biofilm formation and removing preformed biofilms, which makes them promising agents that could be used to develop more effective treatment solutions [96].

The Table 4 summarizes the advantages and disadvantages of the new strategies to control biofilm-associated ocular infections.

## 7. Synergistic Therapy against Biofilms Involved in Ocular Infections

A further approach to the treatment of fungal infections, which are difficult to eradicate, is combination therapy. Drug combinations are a suitable option in specific situations. For the treatment of fungal infections, monotherapy is often indicated, but evolution is not always favorable. The failure of treatments can be due to variations in the bioavailability of the drug between different body tissues. Another problem is the formation of biofilms that limit the penetration of some antimycotic drugs.

Breit. et al. reported that a greater improvement in eye infections was achieved in cases of *C. albicans* and *C. glabrata* endophthalmitis when six patients were treated first with fluconazole and then, subsequently, with a combination of orally administered drugs and intravitreal or i.v. (e.g., voriconazole and caspofungin). Administration of fluconazole together with systemic amphotericin B deoxycholate or, in advanced disease, intraocular amphotericin B deoxycholate and systemic fluconazole represents a therapy with great results [97]. Recently, it was observed, that in cases of keratitis, a combination of topical antifungals amphotericin B with natamycin and voriconazole followed by amphotericin B can be used [98]. Previous studies have shown that the administration of natamycin and voriconazole together has a greater effectiveness than individual treatments, showing a synergistic or additive action in some species of *Fusarium* spp. [99].

Díaz-Tomé et al. [100] developed new formulations of natamycin and voriconazole to treat FK in the presence of resistant species. The formulations were designed to ensure topical administration and confirmed that the combined action of the two antifungals was effective on the test species. The advantages of combination therapy include a greater spectrum of action, a faster effect, possible synergy, a short duration, a delay in the development of resistant species and greater coverage in the case of mixed infections. There are also disadvantages, including high costs, adverse reactions, and a possible antagonistic action between the treatments used.

## 8. Conclusions

Fungal eye infections continue to be the main cause of eye disease. *Candida* spp., *Aspergillus* spp., and *Fusarium* spp. are the most common pathogens responsible for corneal infections and mycosis of the eyeball. So, fungal eye infections represent a medical emergency. Indiscriminate drug use and acquired immunodeficiency syndrome have contributed to the increase in these infections. Fungal infections are difficult to treat, mainly due to the ability of fungi to form mono- and poly-microbial biofilms and the resulting increased resistance to conventional antifungal treatments. Early detection and timely therapy have a significant impact on the course of the disease and can reduce complications (e.g., blindness). In this review, we highlight the efficacy of new drug formulations, including direct drug delivery to the infected region to manage cases of candida ocular biofilm by providing a delayed release of the antimicrobial agents, overcoming the difficulty of limited penetration, and reducing epithelial toxicity. Future focused research activities must elucidate the role of these new formulations also in combination with pre-existing drugs on ocular *Candida* biofilm through in vivo and ex vivo studies.

## Figures and Tables

**Table 1:** Sites, etiological agents, risk factors, symptoms, and signs associated with candida eye infections.

Infection	Sites	Most Frequent Etylogical Agents	Risk Factors or Comments	Symptomps and Signs
Keratitis	Cornea	*C. albicans*, *C. parapsilosis*, *C. viswanathii*	- Ocular disease (e.g., insufficient tear secretion, defective eyelid closure). - Systemic disease (e.g., diabetes mellitus, immunosuppression). - Epithelial defect due to herpes keratitis or contact lenses.	- Foreign body sensation. Vision loss, sensitivity to light, slow onset of increasing pain. - Purulent discharge, conjunctival hyperemia, corneal epithelial defects, stromal infiltrate, anterior chamber reaction, and hypopyon.
Choroiditis/ Chorioretinitis	Choroid +/− Retina	*C. albicans*, *C. parapsilosis, C. viswanathii*, *C. glabrata*	Immunocompromised, drug addicts, intravenous catheters, corticosteroids, parenteral or broad-spectrum antibiotic therapy during septicemia.	- Asymptomatic or reduced vision in the case of macular involvment - Multiple, bilateral, white, well-circumscribed chorioretinal lesions less than 1mm in diameter, vascular sheathing, intraretinal hemorrhages.
Endophthalmitis	Anterior chamber, Vitreous, Retina, Choroid	*C. albicans*, *C. parapsilosis*, *C. viswanathii*, *C. dubliniensis*, *C. glabrata*	- Exogenous: post-trauma, post-surgery, and post-keratitis. - Endogenous: immunocompromised, drug addicts, intravenous catheters, corticosteroid, parenteral or broad-spectrum antibiotic therapy during septicemia.	- Severe eye pian redness of the sclera, sensitivity to light, reduced vision. - Chorioretinal lesion. Vascular sheathing. Intraretinal haemorrhages. - Vitritis with characteristic exudates with string of pearl appearance and inflammation of the anterior segment.

**Table 2:** Diagnosis of certain eye disorders.

Eye Disease	Diagnosis
Keratitis	- Etiological diagnosis can be performed by corneal scraping, followed by culture in liquid or solid medium or microscopic analysis. - Molecular methods may also be used for etiological analysis (e.g., PCR). - Corneal biopsy: it is possible to perform when corneal scrapings yield negative results. - In vivo, a new noninvasive diagnostic tool is confocal microscopy.
Ocular candidiasis	- Etiological diagnosis consists of cultures exams. The biological specimen should be collected by aqueous or vitreous sampling. - A quick and presumptive diagnosis can be performed by direct microscopic examination. - In the case of endogenous endopthalmitis, it is recommended to execute blood cultures. - Detection of *Candida* DNA in intraocular fluid can be performed using PCR assays.

**Table 3:** Current treatment of *Candida* eye infections.

	Treatment	Comments
*Candida* Keratitis	Topical natamycin 5% Topical amphotericin B 0.15–0.3% Topical voriconazole 1%	- Consider the addition of systemic treatment (oral azoles) in the case of severe disease and/or immunocompromised patients. - Cycloplegics and antibiotics for the management of pain and complications caused by bacterial infections. - Keratoplasty in the presence of perforation or ineffectiveness of therapy.
*Candida* Choroiditis	Systemic amphotericin B	The lesions are external to the BRB, so amphotericin B, which is effective against *Candida* but poorly cross the BRB, is indicated.
*Candida* Chorioretinitis	Systemic fluconazole (oral or intravenous)	The lesions are internal to the BRB, so a drug which has demonstrated crossing the BRB is necessary.
*Candida* endophthalmitis	Intravitreal amphotericin B plus systemic fluconazole +/− Vitrectomy	Vitrectomy is useful both as a diagnostic and as a therapeutic tool.

**Table 4:** Advantages and disadvantages of the new strategies to control biofilm-associated ocular infections.

Treatment	Advantages and Disadvantages
FLZ-polymeric nanoparticle formulation	Higher antifungal activity compared to the pure drug.
MET nanoparticles	MET penetrates within biofilms and MET-AmB eye drop formulations may provide a superior therapeutic outcome in biofilm-associated ocular fungal infections. MET-AmB formulation was a more active antifungal formulation when compared to the drug alone.
Posaconazole (PSC)	Broad-spectrum antifungal action. Highly lipophilic molecule, insoluble in water, and a relatively high-molecular-weight compound, which is likely to limit its ocular bioavailability. A novel delivery system of PSC-micelles presents safe anti-fungal activity, which providse delivery via the ocular route.
Metal-based anti-infective agents	Antimicrobial properties. Should be used with caution because of their toxicity.
Lyticase and gallium ions co-integrated nanoparticle (MLPGa)	MLPGa can degrade exopolysaccharides and act on planktonic cells and mature biofilms of *Candida*, without causing adverse effects.
Olaminosomes	Olaminosomes improve the corneal penetration and antifungal efficacy of fenticonazole nitrate.
*Orobanche crenata* (OCLE)	Fungistatic effect.
Cannabinoids	Cannabinoids show side effects, especially when administered systemically. Nanostructures composed of hydrophobic materials seem to be the best option to carry cannabinoids due to their lipophilic character. CBD has a good effect on inhibiting biofilm formation and removing preformed biofilms.
Coated contact lenses	Coated contact lenses with ternary multifunctional hybrid nanocoatings are designed for the treatment of bacterial and fungal keratitis.

## Data Availability

Not applicable.
